# Syringomyelia

**Published:** 1898-03

**Authors:** 


SYRINGOMYELIA. An Essay to which was Awarded the Alvarenga Prize of the College of Physicians of Philadelphia, for the Year 1895. By GUY HINSDALE, A. M., M. D., Fellow of the College of Physicians of Philadelphia, etc. Philadelphia: P. Blakiston Son & Co. 1897.

This monograph is a careful study of the subject as regards its etiology, pathology and symptomatology. The author has made his subject clear by good illustrations and diagrams. He presents two hitherto unpublished cases. The appended bibliography is very complete, there being 514 references to 388 authors.

